# The role of correlates of protection in overcoming barriers to vaccine development and demonstrating efficacy

**DOI:** 10.1038/s41541-024-00873-5

**Published:** 2024-04-13

**Authors:** Deborah F. King, Helen Groves, Charlotte Weller

**Affiliations:** Infection Disease Strategic Programme, Wellcome, 215 Euston Road, London, UK

**Keywords:** Infectious diseases, Vaccines

## Barriers to vaccine development

Vaccine development requires the conduct of clinical trials to generate the necessary safety, quality and efficacy data needed for licensure^1^. Starting initially with small trials to demonstrate safety, and progressively scaling up to look at immunogenicity and finally efficacy, this process has resulted in the development of vaccines against 25 diseases, with more in the pipeline^2^ which have significantly reduced morbidity and mortality since their introduction^3^.

Vaccine development takes on average 10–15 years and costs at least $500 m to bring a new product to market, with probability of success estimated to be as low as 10%^4^ The high level of investment of both time and money needed to progress vaccine candidates combined with high risk of failure can disincentivize development of some products. The feasibility of clinical development and the likelihood of regulatory approval are key drivers of developer decision-making when considering which products to progress, especially as vaccines move towards pivotal efficacy studies which require significant investment^5^. Whilst quality and safety standards are well defined, establishing efficacy can be challenging.

Given that infectious diseases still kill millions of people worldwide, particularly in Africa, some parts of south-east Asia and south America^6^ alternative strategies must be considered to address the challenges of progressing vaccines through late-stage development.

These challenges can be technical, such as low disease incidence, diagnostic or enrolment challenges, or lack of understanding of protective immunity in target populations. Challenges can also be financial, with candidate vaccines failing to attract the necessary level of investment to conduct efficacy trials if market demand or return on investment are uncertain, falling into “the second valley of death” between late-stage clinical development and licensure^7^.

The majority of vaccines licenced to date have been assessed for efficacy against clinical disease endpoints in randomised controlled trials (RCT’s), this approach is viewed as the gold standard method to demonstrate the efficacy data required by regulators for licensure. However, this traditional approach is not always feasible in certain situations.

The outbreak of Sudan ebolavirus (SUDV) in Uganda in 2022 highlights this issue for emerging infectious diseases. When the outbreak of SUDV was declared in Uganda^8^, there were no licenced vaccines, but there were 3 vaccine candidates in development^9^ Plans were rapidly drawn up by Ugandan authorities and the WHO, to conduct a “ring vaccination” study using WHO’s SOLIDARITY trials core protocol to assess the effect of a single vaccine dose in protecting recent contacts of newly confirmed cases of SUVD against lab-confirmed SUVD^10^ The first doses of candidate vaccines arrived in Uganda just 79 days after the outbreak was declared. However, before the trial started, the outbreak was declared over. Given the deadly nature of ebolavirus disease (this outbreak recorded 142 confirmed cases and 55 confirmed deaths) medical counter measures to control outbreaks are still urgently needed. Whilst the control and eventual ending of the outbreak was achieved through leadership, teamwork, contact tracing testing and control measures such as quarantines and lockdowns, such measures are not without their significant downsides, particularly for the poorest people in societies where not working means no income to support their families^11^.

This issue does not only affect emerging infectious diseases, other examples include:

- Where disease incidence is low e.g. a cluster-randomised ring vaccination trial for a Nipah virus vaccine was estimated to take 516 years and over 163,000 vaccine doses under current epidemic conditions^12^.
- Where large trials are required e.g. licensure of a maternal GBS vaccine to prevent neonatal disease require enrolment of up to 80,000 pregnant women^13,14^, or prevention of enteric fever caused by S. paratyphi A, where low attack rates mean efficacy studies would require 100,000–250,000 participants^15^.
- Where unpredictable market demand and return on investment mean products fail to attract necessary investment e.g. new TB vaccines^16,17^.

## The need to understand protective immunity: the case for correlates of protection

Identification of correlates of protection, (immune responses associated with protection from disease), that can act as *predictors of efficacy* has the potential to unlock the development of safe, technically promising and potentially life-saving vaccines. Supporting research into discovery and use CoP data has the potential to improve go/no-go decision making in clinical development, allow rationale design of new or improved vaccines, reduce the time and cost of phase 3 testing by informing clinical trial design and provide a pathway to continue development when clinical efficacy studies are unfeasible. Coupling CoP-based approaches with post-authorisation studies to demonstrate effectiveness could lead to licensure when clinical efficacy cannot be feasibly achieved.

## Barriers to the use of correlates of protection, and solutions: The 4 C’s

In September 2022 Wellcome convened vaccine developers, regulators and policy makers to determine how to embed the identification and validation of correlates of protection early in the clinical development process and enable their use throughout.

Gaps in consistency of data collection and analysis, lack of collaboration and co-ordination between stakeholders and clear communication of evidence gaps and how to address these were all identified as challenges (workshop report in press).

## Conclusions

Licenced vaccines have traditionally been designed empirically, and achieved authorisation through RCT’s based on clinical endpoints to demonstrate efficacy. However, such approaches are not always feasible and have not been successful against more technically challenging targets such as HIV. Alternative approaches to inform rationale vaccine design, demonstrating effectiveness and increasing probabilities of success are needed to stop new products stalling in development, and leaving vulnerable populations at risk of morbidity and mortality caused by infectious diseases.

Developing new methods to define protective immune responses, coupled with commitment to post-introduction studies to ensure safety and effectiveness, has the potential to improve our understanding of protective immunity in target populations which in turn will inform vaccine design, development and use.

Without new approaches to develop vaccines, many communities will continue to bear the burden of infectious diseases and are exposed to the risk of infectious diseases escalating. The COVID-19 pandemic reminded us all of the devastating impact infectious diseases can have on our lives, and whilst multiple vaccines were rapidly developed for this disease, new vaccines are still needed to prevent death and disability caused by endemic diseases such as TB, and to be prepared for future outbreaks. Given the growing threats to controlling infectious diseases such as climate change and antimicrobial resistance, it is important to act now to ensure that we are better prepared to tackle infectious diseases that affect everyone now and in the future.
